# Psychological factors associated with headache frequency, intensity, and headache-related disability in migraine patients

**DOI:** 10.1007/s10072-021-05453-2

**Published:** 2021-07-25

**Authors:** Theresa Klonowski, Peter Kropp, Andreas Straube, Ruth Ruscheweyh

**Affiliations:** 1 Department of Neurology, Ludwig-Maximilians-Universität, Klinikum Grosshadern, Munich, Germany; 2 Institute of Medical Psychology and Medical Sociology, University Medical Center Rostock, Rostock, Germany

**Keywords:** Migraine, Psychological factors, Catastrophizing, Depression, Social avoidance, Disability, Frequency

## Abstract

**Background:**

Several psychological cofactors of migraine have been identified, but relationships to different headache parameters (e.g., headache frequency vs. headache-related disability) are only incompletely understood.

**Methods:**

We cross-sectionally assessed 279 migraine patients at their first presentation at our tertiary headache center. We obtained headache and acute medication frequency, pain intensity, the Migraine Disability Assessment Scale (MIDAS), and the Pain Disability Index (PDI) as headache-related outcomes as well as scores of the Hospital Anxiety and Depression Scale (HADS), the Pain Catastrophizing Scale (PCS), Pain-Related Control Scale (PRCS), and Avoidance Endurance Questionnaire (AEQ) as psychological factors.

**Results:**

Linear regression models revealed the highest associations of the psychological factors with the PDI (adjusted *R*^2^ = 0.296, *p* < 0.001, independent predictors: PCS, AEQ social avoidance, depression) followed by the MIDAS (adjusted *R*^2^ = 0.137, *p* < 0.001, predictors: depression, AEQ social avoidance) and headache frequency (adjusted *R*^2^ = 0.083, *p* < 0.001, predictors: depression, AEQ humor/distraction). Principal component analysis corroborated that psychological factors were preferentially associated with the PDI, while the MIDAS loaded together with headache frequency.

**Conclusion:**

Our results suggest that psychological factors are more strongly associated with the subjective degree of headache-related disability measured by the PDI than with the days with disability (MIDAS) or the more objective parameter of headache frequency. This once again highlights the need for comprehensive assessment of migraine patients with different headache parameters and the need for considering psychological treatment, especially in patients with high disability.

**Supplementary Information:**

The online version contains supplementary material available at 10.1007/s10072-021-05453-2.

## Introduction

Migraine is a primary headache disorder affecting ~ 10–15% of the population [1, 2] and associated with a high burden of disease [3] and relevant socioeconomic costs [4].

Just like other forms of chronic pain, migraine has significant psychological cofactors [5]. The biopsychosocial model of pain applied to headache addresses the complex interactions between biological, psychological, and social factors in the development and maintenance of headache [6]. Psychological factors include affective, cognitive, and behavioral aspects, which influence the patient´s perceptions of and responses to headache and may contribute to the large individual differences in pain expression.

Headache always involves negative affects [7]. Subclinical anxiety or depression symptoms are associated with migraine, in particular with migraine-related disability [8]. There is also a high comorbidity of migraine and both affective and anxiety disorders [9]. Directions and mechanisms underlying these associations are still poorly understood. A systematic review [10] proposes a complex bidirectional association between anxiety and depression symptoms and migraine. The risk of developing depression is increased in migraine patients and vice versa.

Regarding cognitive aspects of migraine, pain catastrophizing describes excessively negative cognitions towards pain and is thought to contribute to increased pain intensity, disability and psychological distress [11]. In migraine, catastrophizing correlates with headache-related disability. A possible relationship to chronicity needs further evaluation [8]. However, self-efficacy, i.e., beliefs of control over pain, achieves a decrease of the negative impact of stress on headache[12].

Regarding behavioral factors, the avoidance-endurance model is well established for musculoskeletal pain and assumes that physical and social avoidance as well as endurance behaviors potentially worsen chronic pain [13]. In migraine, avoidance and endurance behavior during and between attacks play an important role in psychological migraine treatment [14]. First results obtained with the AEQ in migraine patients suggest that avoidance (especially social avoidance) is related to disability, while a negative impact of endurance behavior could not be confirmed [15].

While the relationship of psychological factors with different headache parameters has been investigated for the single factors as described above, an integrative view determining the most relevant associations across these factors is currently missing.

Assessing migraine severity requires a multifactorial approach. Clinically, the amount of headache days per month is the most frequently used parameter. However, headache-related disability is essential to capture the impact on the patient’s daily life [16]. Guidelines have recommended to not only assess headache and acute medication frequency, but headache-related disability and headache intensity, too [17]. Considering the results of previous studies investigating single relationships, we hypothesized that (1) psychological factors were rather associated with the subjective measure of headache-related disability than with the more objective measure of headache frequency. We also hypothesized that (2) psychological factors from all three domains (affective, cognitive, and behavioral) would show independent relationships to headache parameters.

Thus, we investigated the relations between psychological factors from these domains (anxiety and depression, catastrophizing and resourcefulness, avoidance and endurance) and headache-related outcome variables (headache and acute medication days per month, headache intensity and headache-related disability) in 279 migraine patients at our tertiary headache center.

## Methods

### Participants

The present study is a retrospective, cross-sectional analysis based on 279 migraine patients, who were assessed and treated within the interdisciplinary outpatient assessment and treatment program in the Upper Bavarian Headache Center at the Department of Neurology, Munich University Hospital, Germany between March 2012 and October 2019.

For more information about the treatment program, see Ruscheweyh et al. [15]. To participate in said program, the patients’ insurance companies must have entered a contract with our headache center that includes special reimbursement modalities and quality control requirements. Within the program, patients (or, in case of adolescents, both the patient and one of their legal guardians) provide written informed consent for their data to be used for quality control and publication in anonymized form. During the first presentation at the headache center, the patient is required to fill in an assessment of headache parameters and a set of questionnaires (specified below). These data were used for the present study. The patients` first appointment also includes a clinical interview and an examination by a trained headache physician as well as an interview by a trained psychologist. Consent from the local data protection commissioner has been obtained. The study was conducted in accordance with the Declaration of Helsinki.

Inclusion criteria were (1) a minimum age of ≥ 16 years, (2) a diagnosis of migraine according to the International Classification of Headache Disorders (ICHD-2, ICHD-3 Beta and ICHD-3[18] versions were used as they became available over the time span of recruiting), (3) adequate knowledge of the German language, and (4) the ability to fill in the questionnaires digitally.

A total of 327 patients participated in our program and completed the set of questionnaires. Of these, 288 received a migraine diagnosis (ICHD). Nine patients had to be excluded due to missing data in one of the main headache outcome variables, leaving 279 for analysis.

### Questionnaires

The variables “headache days per month,” “headache intensity” (on headache days, range: 0–10, 0 = no pain, 0 = most intense pain imaginable), and “days of consumption of acute headache medication per month” were based on subjective patient reports. Said reports considered the last 3 months and therefore were averaged. The diagnoses “episodic migraine with or without aura” and “chronic migraine” were based on the ICHD-2, ICHD-3 Beta, or ICHD-3 [18] as valid at the time of recruitment. When the ICHD-2 was used for the diagnosis “chronic migraine,” the appendix criteria (A1.5.1) were applied. Medication overuse was defined according to the ICHD as triptan or combination analgesic intake on ≥ 10 days per month, analgesic intake on ≥ 15 days per month or intake of acute headache medication of different classes on ≥ 10 days per month.

#### Migraine disability assessment scale (MIDAS) [19]

The MIDAS measures the headache-related disability within the last 3 months in three areas of life: work/school, household chores, and family/social/leisure activities. Five items assess the number of days with complete unproductiveness (for all three areas), or productiveness reduced by half or more (for the first two areas). The MIDAS score is formed by summing the items and ranges from 0 to 270. The disability can be categorized in four grades: (I) little or no disability (MIDAS score 0–5), (II) mild (6–10), (III) moderate (11–20), and (IV) severe disability (> 21).

#### Pain disability index (PDI) [20]

The PDI is an instrument to measure disability associated with chronic or frequent pain in general (not specifically with headache). It includes seven items that measure the degree to which pain limits the patient in his usual activities (no specific time frame is given) on 10-point scales (from 0 “no disability” to 10 “complete disability”) in seven categories: family/social responsibilities, recreation, social activity, occupation, sexual behavior, self-care, and life-support activities. The PDI score is calculated as the average of the seven items and ranges from 0 to 10.

#### Hospital anxiety and depression scale—German version (HADS) [21]

The HADS is a 14-item self-report measure of anxiety (HADS-A, 7 items) and depression (HADS-D, 7 items) symptoms over the last week. Each item is scored on a 4-point scale (e.g., “I still enjoy the things I used to enjoy.” 0 “definitely as much,” 1 “not quite so much,” 2 “only a little,” 3 “hardly at all”). Some of the items indicate higher anxiety or depression symptoms by lower scale values and therefore have to be reversed before score calculation. The two subscale scores are formed by summing the corresponding items (range: 0–21). Anxiety/depression symptoms are considered mild (score 8–0), moderate (11–14), or severe (≥ 15).

#### Pain catastrophizing scale (PCS)[22]

The PCS asks about feelings and thoughts with respect to pain on a 5-point scale (e.g. “I worry all the time about whether the pain will end.” 0 “not at all” to 4 “all the time”). The 13 items are summed up to a total score (0–52) and three subscales: rumination (4 items), magnification (3 items) and helplessness (6 items). Higher values indicate higher levels of pain catastrophizing. For the purpose of the present study, we specified the PCS for headache by referring to headache in the instructions [23]. Only the total score was used. Clinically relevant pain catastrophizing is defined as PCS > 30.

#### Pain-related control scale (PRCS)[24]

The PRCS consists of 16 items asking about attitudes and reactions towards chronic pain on a 6-point scale (e.g. “I try to forget about the pain.” 0 “not at all”—5 “very much”). It consists of two conceptually separate subscales calculated as averages of the corresponding items: helplessness (PRCS-H, 7 items) and resourcefulness (PRCS-R, 9 items). Scores range from 0 to 5, higher values indicating more helplessness/resourcefulness.

#### Avoidance endurance questionnaire (AEQ)—behavioral subscales[13]

The AEQ assesses cognitive and behavioral responses to mild and severe pain within the last 2 weeks on 7-point scales (e.g., “I stop physical demanding activities.” 0 “never”—6 “all the time”). Here, we only used the four behavioral subscales: avoidance of social activities (ASAS, 6 items), avoidance of physical activities (APAS, 5 items), humor distraction (HDS, 5 items), and pain persistence (PP, 7 items). We did not separately report the behavioral endurance scale (BES), since it is the combination of the HDS and PP scales. Subscale scores are formed by averaging item values, resulting in a range from 0 to 6. For the purpose of the present study, we averaged scores of the mild and severe pain items.

The digital questionnaires did not allow missing data.

### Statistics

Statistical analyses were conducted using the Statistical Package of Social Sciences (SPSS Statistics; IBM, Ehningen, Germany) version 25. All tests were two-tailed. *P* < 0.05 was considered significant.

The following headache outcome variables were used: headache days per month, days with intake of acute headache medication per month, headache intensity, MIDAS, and PDI scores.

#### Zero-order correlations

In a first step, zero-order correlations between headache outcome variables and psychological factors (HADS-A, HADS-D, PCS, PRCS-H, PRCS-R, AEQ-APAS, AEQ-ASAS, AEQ-HDS, AEQ-PPS) as well as zero-order correlations between headache outcome variables and age were computed using Spearman’s rho. Relations to sex were determined using the Mann–Whitney *U* test.

#### Multiple linear regressions

Stepwise multiple linear regressions were calculated in a second step to identify significant independent associations for every headache outcome variable. We used psychological factors that had shown significant zero-order correlations as independent variables. Age was additionally included as an independent variable when significantly correlated with the headache outcome variable (which applied to headache intensity and PDI).

Assumptions of linear regression analysis were tested for each regression model. Based on the results, a sensitivity analysis after excluding 16 possible outliers was performed (see supplementary material).

#### Principal component analysis (PCA)

Results of the regression analysis and correlations between the headache outcome variables suggested that these variables and factors might originate from two separate dimensions (see the “Results” section). To further explore and illustrate this possibility, we performed a PCA with varimax rotation and Kaiser normalization. All headache outcome variables and those psychological variables that were significantly associated to them in regression analyses (HADS-D, AEQ-HDS, AEQ-ASAS and PCS) were included. The Kaiser–Meyer–Olkin measure of sampling adequacy was 0.656 (adequate for PCA[25]). Bartlett’s test of sphericity was significant (*p* < 0.001), indicating that correlations are large enough to calculate a PCA.

## Results

Two hundred seventy-nine migraine patients were included (62.4% episodic migraine, 37.6% chronic migraine). Demographic and headache characteristics and questionnaire results are shown in Table 1.

**Table 1:** Characteristics of the study population (*n* = 279)

Age *[years]*	36.9 ± 12.7 (range: 16 – 71)
Sex	235 females (84.2%)
Episodic migraine	174 (62.4%)
- Without aura [n(%)]	128 (45.9%)
- With aura [n(%)]	46 (16.5%)
- No medication overuse [n(%)]	141 (81%)
- Medication overuse [n(%)]	33 (19%)
Chronic migraine [n(%)]	105 (37.6%)
- No medication overuse [n(%)]	61 (58.1%)
- Medication overuse [n(%)]	44 (41.9%)
*Headache outcome variables*	
Headache days per month	13.5 ± 8.2
Days with intake of acute headache medication per month	8.4 ± 6.1
Headache intensity *[0–10]*	6.9 ± 1.6
MIDAS score *[0–270]*	48.7 ± 42.0
MIDAS grade	
- I [n(%)]	12 (4.3%)
- II [n(%)]	19 (6.8%)
- III [n(%)]	42 (15.1%)
- IV [n(%)]	206 (73.8%)
PDI score *[0–10]*	4.4 ± 2.1
*Psychological factors*	
HADS-anxiety *[0–21]*	7.6 ± 3.9
HADS-depression *[0–21]*	5.0 ± 3.8
PCS score *[0–52]*	26.0 ± 10.8
PRCS-helplessness *[0–5]*	2.2 ± 0.9
PRCS-resourcefulness *[0–5]*	2.3 ± 0.7
AEQ-APAS (Avoidance of physical activities) *[0–6]*	3.5 ± 1.0
AEQ-ASAS (Avoidance of social activities) *[0–6]*	2.8 ± 1.1
AEQ-HDS (Humor Distraction) *[0–6]*	2.7 ± 1.0
AEQ-PPS (Pain Persistence) *[0–6]*	3.5 ± 0.9

Mean and standard deviation or percentages are given. *MIDAS* Migraine Disability Assessment Scale, *PDI* Pain Disability Index, *HADS* Hospital Anxiety and Depression Scale, *PCS* Pain Catastrophizing Scale, *PRCS* Pain-Related Control Scale, *AEQ* Avoidance Endurance Questionnaire, *n* = frequency

In our sample, 37.7% met the criterion for clinically relevant pain catastrophizing (PCS > 30).

Regarding the HADS-A score, 28.3% of the patients showed mild, 20.4% moderate, and 3.2% severe anxiety symptoms. In the HADS-D, 16.1% showed mild, 6.1% moderate, and 2.9% severe depression symptoms. These results indicate that 52.0% of our sample showed anxiety and 25.1% showed depression symptoms.

### Correlations between psychological factors and headache outcome variables

Zero-order correlations are given in Table 3 (see Appendix). Correlations were classified as small (rho ≥ 0.1), medium (rho ≥ 0.3) or large (rho ≥ 0.5) according to Cohen [26].

The highest correlations with psychological factors were found for the disability measures, especially the PDI. The PDI showed the largest correlation with pain catastrophizing (rho = 0.413, *p* < 0.001) and medium to small correlations with anxiety and depression, PRCS-helplessness and social avoidance scores. The MIDAS score showed the largest correlations with social avoidance and depression scores (rho = 0.312 and 0.311, both *p* < 0.001) and smaller correlations with anxiety, catastrophizing, and PRCS-helplessness scores. Compared with the PDI, the correlation between MIDAS and PCS was smaller (rho = 0.159, *p* = 0.008).

Correlations were smaller for the remaining headache outcome variables. “Headache days per month” showed small positive correlations with depression scores and the humor-distraction scale of the AEQ. They also showed a small negative correlation with social avoidance, but no significant correlation with the PCS. None of the psychological factors significantly correlated with “acute medication intake days per month.” A medium-sized positive correlation was found between headache intensity and pain catastrophizing (rho = 0.217, *p* < 0.001). Small positive correlations were found with physical and social avoidance and a small negative correlation with PRCS-resourcefulness.

PDI (rho = 0.218, *p* < 0.001) and headache intensity (rho = 0.122, *p* = 0.042) were significantly correlated with age, while “headache days per month” (rho =  − 0.079, *p* = 0.187), “acute medication intake days per month” (rho = 0.066, *p* = 0.272), and MIDAS scores (rho =  − 0.048, *p* = 0.428) were not. There were no significant differences between male and female subjects in any of the headache outcome variables: “headache days per month” (*Z* =  − 0.741, *p* = 0.459), “acute medication intake days” (*Z* =  − 1.597, *p* = 0.110), headache intensity (*Z* =  − 0.597, *p* = 0.550), MIDAS score (*Z* =  − 0.994, *p* = 0.320) and PDI (*Z* = -0.176, *p* = 0.860).

### Linear regression analyses

A multiple linear regression analysis was performed for each headache outcome variable to detect independent associations with those psychological factors, which had shown significant zero-order correlations, and with age (where significant, see Table 4, Appendix).

The highest amount of variance (adjusted *R*^2^ = 29.6%) was explained for the PDI. Pain catastrophizing, social avoidance, depression, and age were significant, independent and positive correlates.

A significant amount of variance (13.7%) was also explained for the MIDAS score, with depression and social avoidance scores being significantly and positively related.

A smaller percentage of variance (8.3%) was explained for the number of headache days per month and significantly related to higher depression and humor distraction scores.

Acute medication intake frequency did not show significant zero-order correlations. Therefore, no model was calculated.

Headache intensity was only associated with higher catastrophizing scores (explained variance 3.3%).

The sensitivity analysis performed after the exclusion of 16 outliers (see statistics section) had very similar results (see Supplementary Table).

### Correlations between headache outcome variables and principal component analysis

Looking at the results above, it is noteworthy that the PCS showed a significant independent association with the PDI score and headache intensity, but not with the MIDAS score, headache or medication days. Also, the examination of correlations between the headache outcome variables revealed that there were significant correlations between the MIDAS score and headache days per month as well as medication days per month (rho = 0.495 and 0.285, both *p* < 0.001) but not between the PDI and headache or medication days per month (rho =  − 0.021, *p* = 0.728; rho = 0.059, *p* = 0.327). In contrast, headache intensity had a medium-sized correlation with the PDI (rho = 0.285, *p* < 0.001), but only a small correlation with the MIDAS (rho = 0.129, *p* = 0.031). These observations suggested that there might be two separate dimensions among the headache outcome variables included in the present study: headache and medication days per month and the MIDAS score on the one side and headache intensity and the PDI score on the other side, with the PCS being more related to the second than the first dimension. We further explored this possibility by calculating a principal component analysis (PCA) including the five headache outcome variables and those psychological factors showing significant relations in the regression models.

There were three components with eigenvalues > 1. The eigenvalue of the third factor was barely above 1 (1.07) and examination of the scree plot supported a two-factor solution. Therefore, we extracted two factors, explaining 47.5% of the total variance. This model also provided the most interpretable solution. The AEQ-HDS values were inverted for this analysis, since it was the only variable that loaded negatively onto the first component.

**Fig. 1:**
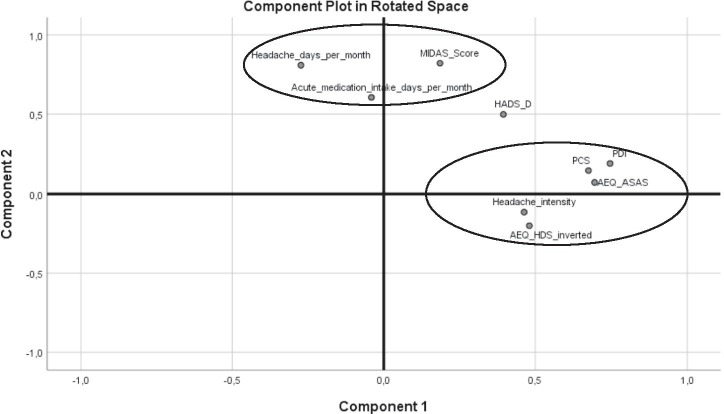
Principal component analysis: component plot in rotated space. Illustration of the factor loadings of the different headache outcome variables and psychological factors onto the two components extracted by principal component analysis. Only those psychological factors showing significant relations with the headache outcome variables in the regression models were included. *AEQ-ASAS* Avoidance Endurance Questionnaire–Avoidance of social activities scale. *AEQ-HDS* Avoidance Endurance Questionnaire–Humor/distraction scale. *HADS-D* Hospital Anxiety and Depression Scale–Depression subscale. *MIDAS* Migraine Disability Assessment Score. *PCS* Pain Catastrophizing Scale. *PDI* Pain Disability Index

The factor loadings of the final PCA model are shown in Table 2 and in Fig. 1. Indeed, headache days per month, acute medication intake days, and MIDAS scores load together onto one component, while PDI and headache intensity load onto the other component. Among the psychological factors, AEQ-ASAS, PCS, and AEQ-HDS (inverted) load onto the same component as PDI and headache intensity while HADS-D loads similarly onto both components.

**Table 2:** Principal component analysis: rotated component matrix

	Component	
	1	2
PDI	**0.747**	0.192
AEQ-ASAS (avoidance of social activities)	**0.696**	0.073
PCS (pain catastrophizing)	**0.676**	0.147
AEQ-HDS (humor distraction, inverted)	**0.480**	− 0.201
Headache intensity [0–10]	**0.463**	− 0.115
MIDAS Score	0.186	**0.821**
Headache days per month	− 0.273	**0.809**
Acute medication days per month	− 0.041	**0.606**
HADS-D (depression)	**0.395**	**0.499**

Factor loadings of the different headache outcome variables and psychological factors onto the two components extracted by principal component analysis. Only those psychological factors showing significant relations with the headache outcome variables in the regression models were included. Factor loadings > 0.3 are marked in bold. *AEQ-ASAS* Avoidance Endurance Questionnaire–Avoidance of social activities scale. *AEQ-HDS* Avoidance Endurance Questionnaire–Humor/distraction scale. *HADS-D* Hospital Anxiety and Depression Scale–Depression subscale. *MIDAS* Migraine Disability Assessment Score. *PCS* Pain Catastrophizing Scale. *PDI* Pain Disability Index

## Discussion

Our results show that (1) psychological factors are more strongly associated with headache-related disability than with headache frequency. (2) Within headache-related disability, the PDI and MIDAS reflect two different dimensions. (3) Depression, catastrophizing, and social avoidance are the psychological factors most strongly associated with headache.

Thus, the results confirm our hypothesis of a stronger association of psychological factors with the subjective parameter of headache-related disability than with the more objective parameter of headache frequency. Similarly, (subjective) patient-reported migraine-outcomes (e.g., quality of life and disability) and treatment satisfaction show only a moderate correlation with objective diary-based data [27, 28]. This gap may be the result of the patients´ estimation, which does not follow a linear function of objective measures and is likely influenced by the patient’s psychological state. Indeed, it has been reported that anxiety and depression influence the relationship between headache frequency and quality of life [29]. However, it must be kept in mind that headache frequency is also not a completely objective parameter since the threshold of what is perceived as headache may vary individually. Moreover, if not assessed by daily completion of a headache diary, headache frequency is also subject to recall bias, which may introduce additional subjective perception into this parameter. Nonetheless, in summary, there is a differential but complex relationship between the more objective and more subjective headache parameters, with subjective parameters being more tightly linked to psychological factors.

Our data also show that the type of headache-related disability considerably differs between PDI and MIDAS. The MIDAS measures the *number of days* with disability whereas the PDI captures the *degree* to which a patient feels disabled. Consequently, in the PCA, the MIDAS loaded together with headache days per month. In contrast, the psychological factors all loaded together with the PDI (except for depression, which loaded onto both factors). This shows that the *degree* to which a patient is not able to perform an activity is more tightly linked to psychological factors than the *number of times* he or she is not able to perform the activity. Headache intensity, which also measures a *degree*, also loaded together with the PDI. In conclusion, the PDI seems to measure a more subjective dimension of disability linked to psychological cofactors whereas the MIDAS (asking patients to count the days with disability) is a more objective instrument. It has to be considered that measuring subjective disability may be more relevant for the patient and better reflect the patient’s total state, even if objective measures generally are considered the primary choice when measuring headache outcomes.

From the different dimensions of psychological factors assessed, in the present study (1) depression (affective), (2) catastrophizing (cognitive), and (3) social avoidance (behavioral) showed the strongest associations with migraine outcome variables (while anxiety, resourcefulness and endurance were less related).

1. How can the relation between migraine and depression be explained? People who suffer from depression often find themselves in a downward spiral of low mood, low energy, a loss of interest and pleasure[30], fewer activities and social withdrawal. Migraine can favor this development, e.g., by leading to reduced social contacts due to fear of headache (see Lewinsohn’s social reinforcement theory of depression [31]) and by activating negative emotions or cognitions that further augment depressive symptoms [32]. According to Beck’s cognitive theory of depression [33], depression is characterized by dysfunctional, negative views of the world, oneself, and the future. These cognitive distortions could in turn lead to a more negative estimation of the headache-related disability. In addition, sleep disturbances are frequent in both depression and migraine and may function as an exacerbating factor for both [34]. The cognitive mediation hypothesis states that the relationship between pain frequency and depression is mediated by coping strategies such as distraction or catastrophizing (see below) [32]. The bidirectional relationship of migraine and depression may also be sustained by shared vulnerability factors like increased activation of the hypothalamic-pituitary adrenal axis (HPA) by stress [10].
  Consistent with previous findings in migraine patients [35], anxiety symptoms were more prevalent (52.0%) than depression symptoms (25.1%) in our sample. Depression but not anxiety scores, however, showed an independent relation to headache-related disability in linear regression. Thus, the present and previous data suggest that while anxiety may be an antecedent or vulnerability factor for the development of migraine [5, 10, 36], depression is more associated with the severity of migraine.
2. In our data set, pain catastrophizing was associated with headache-related disability (PDI) and headache intensity but not with headache frequency. This is consistent with previous studies in headache reporting a tighter association of catastrophizing with disability and quality of life than with objective pain characteristics or physical impairment [11, 37]. Again, bidirectional associations are plausible: catastrophizing demands time as well as cognitive and emotional resources and may lead to a stress reaction, which in turn can have a negative effect on migraine severity [38]. Sullivan proposes that the appraisal-related process of catastrophizing directly affects pain experience [39]. Nonetheless, it is plausible that patients experiencing more severe pain develop a more extensive fear of pain and catastrophizing [40].
  Although pain catastrophizing was found to be a risk factor for the development of chronic pain in several pain disorders (e.g., postoperative pain, joint pain and low back pain [22]), our and several previous studies found neither an association of catastrophizing with chronic (vs. episodic) migraine nor with headache frequency [8, 37]. This might partially be due to the fact that higher migraine frequencies (especially within the range of chronic migraine) are often associated with lower headache intensity [18] a fact that might also be confounded by the effects of medication overuse. According to our results, lower headache intensity is linked to less catastrophizing. Furthermore, it must be noted that chronic migraine is defined primarily by headache frequency (≥ 15 headache days per month for at least 3 months [18]). At this point, it differs from the definition of chronicity in other pain disorders (pain duration of ≥ 3 or ≥ 6 months [41]).
  In contrast to the large correlations between headache outcome variables and catastrophizing, the small correlations with resourcefulness did not survive linear regression. Similarly, in a previous study catastrophizing and external but not internal locus of control were related to headache outcome variables [8]. This suggests that dysfunctional coping strategies are more powerful in migraine than functional strategies. However, it has to be considered that both studies were conducted at tertiary care centers. Maybe the effect of resourcefulness is larger in less severely affected patients.
3. On the behavioral level, in our sample, average physical avoidance scores were higher than social avoidance scores. Nevertheless, only social avoidance was associated with headache outcome variables. Once more, this is likely to be a bidirectional relationship, with migraine inducing social avoidance as described above. However, social avoidance can also lead to increased sensitivity to stimuli like noise, visual disturbance or stress [42]. The relation to disability might be explained using Lewinsohn’s social reinforcement theory and depression as a mediator [31]. Moreover, the definition of headache-related disability includes reduced or disabled social contacts [19, 20]. It is surprising that social avoidance is not related to headache frequency, since one might hypothesize that less frequent headaches evoke less avoidance behavior. There were fewer associations between headache outcome variables and endurance. In the factor analysis, smaller scores on the humor-distraction endurance scale loaded with higher PDI scores and higher pain intensities. Similarly to our previous results [15], this suggests that endurance behavior does not have negative consequences in migraine, at least not at group level.

In conclusion, our results indicate that migraine patients should be assessed by a comprehensive range of different headache parameters. Furthermore, psychological treatment should especially be considered in patients with a high level of disability and the factors depression, catastrophizing and social avoidance particularly be taken into account.

### Strengths and limitations

A limitation in our study is the fact that all data come from tertiary care patients, precluding direct extrapolation to other populations. However, one could argue that psychological cofactors may be especially meaningful in severely affected patients. Instead of the headache-specific instrument HIT-6 (headache impact test) for the assessment of disability we used the PDI due to budget restrictions. For an analysis of the difference between instruments assessing the *number of days* with disability and the *degree* of disability, the PDI might be even more adequate than the HIT-6. This is because the HIT-6 asks “how often have you felt [disabled]” on a scale from “never” to “always,” which seems more similar to what is assessed by the MIDAS.

In addition, the validated measures we used refer to different recall periods (e.g., MIDAS past 3 months, AEQ 2 weeks, HADS 1 week). This might have artificially reduced correlations. Furthermore, assessment of headache frequency and intensity was based on subjective reports, not on headache diaries. Due to quick patient scheduling, only a minority of patients is able to present a complete 3-months headache diary at the first visit at our center. This introduced recall bias and additional subjective perception into the parameter of headache frequency, reducing the distinction between objective (headache frequency) and subjective (disability) headache outcome variables. The most important strength of the present study is that it provides an integrative view of the relationship between psychological cofactors from several dimensions (affective, cognitive, behavioral) and clinically relevant headache outcome variables (frequency, intensity, two different measures of disability), in a suitably large population of migraine patients. Finally, it must be considered that the present data are cross-sectional. It will be an interesting follow-up project to investigate the relation of psychological factors to the *change* in headache days and disability over treatment and see if this also reveals two different dimensions.

### Summary

The present data show that headache outcome variables as well as headache-related disability have several dimensions, which can be roughly divided into two groups: (1) The first dimension consists of headache frequency, acute medication frequency and days with disability (MIDAS), with limited relation to psychological factors. (2) The second dimension includes the degree of disability (PDI) and headache intensity, with extensive relation to psychological factors. The psychological factors independently related to headache outcome variables were depression, catastrophizing and social avoidance.

### Supplementary Information

Below is the link to the electronic supplementary material.

Supplementary file1 (PDF 87 KB)

## Data Availability

Raw data are available from the corresponding author on reasonable request.

## Appendix

**Table 3:** Zero-order correlations between headache characteristics, headache-related disability and scores of psychological factors (*n* = 279)

	HADS-A (Anxiety) (*n* = 279)	HADS-D (Depression) (*n* = 279)	PCS (Pain catastrophizing) (*n* = 279)	PRCS-H (Helplessness) (*n* = 277)	PRCS-R (Resourcefulness) (*n* = 277)	AEQ-APAS (Avoidance of physical activities) (*n* = 277)	AEQ-ASAS (Avoidance of social activities) (*n* = 274)	AEQ-HDS (Humor/ distraction) (*n* = 274)	AEQ-PPS (Pain persistence) (*n* = 277)
Headache days per month	0.033 *p* = 0.578	**0.186** ***p*** **= 0.002**	-0.004 *p* = 0.952	0.062 *p* = 0.302	0.032 *p* = 0.600	**-0.170** ***p*** **= 0.005**	-0.106 *p* = 0.081	**0.166** ***p*** **= 0.006**	0.066 *p* = 0.271
Days with intake of acute headache medication per month	0.100 *p* = 0.096	0.115 *p* = 0.056	0.062 *p* = 0.305	0.071 *p* = 0.237	-0.038 *p* = 0.528	-0.065 *p* = 0.284	0.003 *p* = 0.961	-0.038 *p* = 0.533	0.000 *p* = 1.000
Headache intensity [0–10]	-0.051 *p* = 0.401	-0.044 *p* = 0.462	**0.217** ***p*** **< 0.001**	0.094 *p* = 0.120	**-0.149** ***p*** **= 0.013**	**0.135** ***p*** **= 0.025**	**0.156** ***p*** **= 0.010**	-0.060 *p* = 0.323	-0.051 *p* = 0.396
MIDAS Score	**0.188** ***p*** **= 0.002**	**0.311** ***p*** **< 0.001**	**0.159** ***p*** **= 0.008**	**0.205** ***p*** **= 0.001**	0.045 *p* = 0.487	0.113 *p* = 0.059	**0.312** ***p*** **< 0.001**	0.040 *p* = 0.467	-0.002 *p* = 0.972
PDI	**0.298** ***p*** **< 0.001**	**0.349** ***p*** **< 0.001**	**0.413** ***p*** **< 0.001**	**0.262** ***p*** **< 0.001**	-0.053 *p* = 0.380	**0.181** ***p*** **= 0.002**	**0.393** ***p*** **< 0.001**	-0.114 *p* = 0.059	-0.061 *p* = 0.310

Spearman’s rho is given (rho ≥ 0.1 was considered small, ≥ 0.3 medium and ≥ 0.5 large). Significant correlations are marked in bold. *p* = probability. *n* = frequency. Number of patients is indicated in parentheses where different from the total n. *MIDAS* Migraine Disability Assessment Scale, *PDI* Pain Disability Index, *HADS* Hospital Anxiety and Depression Scale, *PCS* Pain Catastrophizing Scale, *PRCS* Pain Related Control Scale, *AEQ* Avoidance Endurance Questionnaire

**Table 4:** Prediction of headache outcomes in linear regression models

Model	*R*	Change in *R*^2^	Significance of the model (*p*)	Independent variables	Standardized beta (in final model)	Significance (*p*, in final model)
*Dependent variable: headache days per month (n* = *279)*						
1	0.216	0.047	< 0.001	**HADS-D**	0.234	< 0.001
2	0.299	0.043	< 0.001	**AEQ-HDS**	0.207	< 0.001
-	-	-	-	AEQ-APAS	-	0.246
Total		*R*^2^ = 0.089 *R*^2^ (corr) = 0.083				
*Dependent variable: headache intensity [0–10] (n* = *279)*						
1	0.191	0.036	0.002	**PCS**	0.191	0.002
-	-	-	-	age	-	0.091
-	-	-	-	PRCS-R	-	0.052
-	-	-	-	AEQ-ASAS	-	0.138
-	-	-	-	AEQ-APAS	-	0.175
Total		*R*^2^ = 0.036 *R*^2^ (corr) = 0.033				
*Dependent variable: MIDAS Score (n* = *274)*						
1	0.333	0.111	< 0.001	**HADS-D**	0.296	< 0.001
2	0.378	0.032	< 0.001	**AEQ-ASAS**	0.183	0.002
-	-	-	-	PCS	-	0.842
-	-	-	-	PRCS-H	-	0.291
-	-	-	-	HADS-A	-	0.265
Total		*R*^2^ = 0.143 *R*^2^ (corr) = 0.137				
*Dependent variable: PDI (n* = *274)*						
1	0.428	0.183	< 0.001	**PCS**	0.301	< 0.001
2	0.500	0.067	< 0.001	**AEQ-ASAS**	0.218	< 0.001
3	0.533	0.035	< 0.001	**HADS-D**	0.180	< 0.001
4	0.554	0.022	< 0.001	**age**	0.152	0.004
-	-	-	-	PRCS-H	-	0.814
-	-	-	-	HADS-A	-	0.890
-	-	-	-	AEQ-APAS	-	0.944
Total		*R*^2^ = 0.307 *R*^2^ (corr) = 0.296				

Stepwise selection from independent variables was performed (inclusion criterion: *p* < 0.05; exclusion criterion for previously included variables: *p* > 0.1). Only variables that showed a significant zero-order correlation with the respective dependent variable were included as independent variables in the linear regression analysis. Variables that entered the final model are marked in bold. *n* = frequency. *R*^2^ = determination measurement. *AEQ-HDS* Avoidance Endurance Questionnaire–Humor/distraction scale. *AEQ-ASAS* Avoidance Endurance Questionnaire–Avoidance of social activities scale. *AEQ-APA*S Avoidance Endurance Questionnaire–Avoidance of physical activities scale. *HADS-A* Hospital Anxiety and Depression Scale–Anxiety subscale. *HADS-D* Hospital Anxiety and Depression Scale–Depression subscale. *MIDAS* Migraine Disability Assessment Score. *PCS* Pain Catastrophizing Scale. *PDI* Pain Disability Index. *PRCS-H* Pain-Related Control Scale–Helplessness. *PRCS-R* Pain-Related Control Scale–Resourcefulness

## Declarations

### Clinical implications

- Psychological factors are more strongly associated with headache-related disability than with headache frequency.

- Within headache-related disability, PDI and MIDAS reflect two different dimensions (degree of disability vs. days with disability).

- Pain catastrophizing, depression, and social avoidance are the psychological factors most strongly associated with headache.

- Especially in patients with high headache-related disability, psychological treatment should be considered.
