# Supplementary material for: Psychological factors associated with headache frequency, intensity, and headache-related disability in migraine patients
Source: Neurol Sci. 2021 Jul 25;43(2):1255–66. doi: 10.1007/s10072-021-05453-2 (PMC8789634; doi:10.1007/s10072-021-05453-2)
Supplement: Supplementary file 1 — Supplementary file1 (PDF 87 KB) [file 10072_2021_5453_MOESM1_ESM.pdf]

Supplementary methods

Assumption of linear regression analysis

We checked for (1.) linearity of the association between dependent and independent variables and (2.) homoscedasticity of residuals by analysing the scatterplot showing unstandardized predicted values and studentized residuals. (3.) To test independence of residuals we calculated the Durbin-Watson-statistic (values close to 2 indicating no autocorrelation). (4.) In order to confirm if there was no or little multicollinearity, intercorrelations of independent variables (rho < .7 was defined as no or little multicollinearity) and the variance inflation factor VIF (VIF > 5 indicating a possible multicollinearity) were considered. (5.) Normal distribution of residuals was tested by inspecting the histogram of residuals and the P-P-plot. (6.) Outliers were analysed using studentized deleted residuals, leverages and Cook´s distances. It resulted that assumptions 1-5 were fulfilled for all regression analyses performed. However, the outlier analysis identified 1 to 10 outliers in each of the four regression analyses. We confirmed that these data points corresponded to real values, not data entry errors. To examine the possible influence of these outliers, we performed a sensitivity analysis, after excluding all 16 identified outliers.

Sensitivity analysis

The independent variables showing a significant association were unchanged. Explained variance somewhat increased for the MIDAS score (from 13.7 to 15%) and was basically unchanged for the other parameters. As inspection of the outliers indicated that these corresponded mostly to the patients with the highest MIDAS and acute medication intake

scores, we decided to stick to the original analysis, as these patients, although infrequent, clearly form part of the patients seen at a tertiary headache center.
